# Long COVID-19 alters muscle architecture and muscle-tendon force transmission: a one-year longitudinal study

**DOI:** 10.3389/fphys.2025.1641046

**Published:** 2025-08-25

**Authors:** Leandro Gomes de Jesus Ferreira, Isabella da Silva Almeida, Rochelle Rocha Costa, Gabrielle Vieira Roriz, Jeam Marcel Geremia, João Luiz Quagliotti Durigan, Rita de Cássia Marqueti

**Affiliations:** ^1^ Laboratory of Muscle and Tendon Plasticity, Graduate Program in Rehabilitation Science, Faculdade de Ciências e Tecnologias em Saúde, Universidade de Brasília, Brasília, Brazil; ^2^ Laboratory of Molecular Analysis, Graduate Program in Rehabilitation Science, Faculdade de Ciências e Tecnologias em Saúde, Universidade de Brasília, Brasília, Brazil; ^3^ Graduate Program in Physical Education, Physical Education Department, Universidade de Brasília, Brasília, Brazil; ^4^ Exercise Research Laboratory, Universidade Federal do Rio Grande do Sul, Porto Alegre, Brazil

**Keywords:** fascicular length, pennation angle, muscle ultrasound, muscle-tendon unit, COVID-19 pandemic

## Abstract

**Introduction:**

There are limited studies on the long-term effects of COVID-19 on skeletal muscle morphology and architecture. Therefore, this study aims to address this gap by assessing the effects of prior COVID-19 infection on quadriceps muscle architecture and tendon-aponeurosis complex (TAC) properties over a one-year period, comparing three cohorts: individuals with moderate COVID-19, individuals with severe COVID-19, and a healthy control group.

**Methods:**

Seventy participants were included in the study and allocated to three groups: moderate COVID-19 (n = 22), severe COVID-19 (n = 18), and control (n = 30). Four assessments were conducted over 1 year for the COVID groups. Maximal voluntary isometric (MVIC) knee extension contractions were performed on an isometric dynamometer, with simultaneous ultrasound imaging of the vastus lateralis (VL) and rectus femoris (RF) muscles. Fascicle length (FL) and pennation angle (PA) were obtained at rest and during MVIC, along with TAC displacement. Generalized Estimating Equation models were used to evaluate muscle variables, with “group” and “time” as factors. The model fit was adjusted, with ‘torque’ as a covariate.

**Results:**

Regarding muscle architecture, FL was greater in the severe COVID-19 group during early post-infection assessments for the RF at rest (p = 0.043). Additionally, both COVID-19 groups exhibited longer VL fascicles compared to controls (p = 0.032). TAC displacement was reduced in the severe COVID-19 group (RF: p = 0.008; VL: p = 0.047) compared to control. TAC stiffness did not differ between groups (p = 0.517), but torque production demonstrated an effect on this variable (p = 0.001). Both COVID-19 groups presented reduced PA for the VL at rest (p = 0.012) compared to control. Additionally, torque played a crucial role in influencing PA in both muscles, at rest and during contraction.

**Conclusion:**

Participants with severe COVID-19 exhibited alterations in muscle architecture, which may contribute to persistent muscular weakness even one-year post-infection. The findings underscore the potential role of muscle strength, particularly the impact of torque on TAC stiffness and PA across all groups. Long COVID-19 rehabilitation and exercise physiologists should prioritize quadriceps strengthening strategies to restore muscle architecture and optimize force transmission.

## 1 Introduction

The COVID-19 pandemic, caused by the SARS-CoV-2 virus, has created a global health crisis, affecting millions of individuals worldwide (Rahman et al., 2021; Adil et al., 2021). The disease ranges from mild respiratory symptoms to severe acute respiratory distress (Umakanthan et al., 2020; Heymann et al., 2020). However, beyond its respiratory effects, COVID-19 has shown a strong influence on the musculoskeletal system, contributing to symptoms such as fatigue, muscle weakness, and exercise intolerance (Ali and Kunugi, 2021; Soares et al., 2022; Andalib et al., 2021; Montes-Ibarra et al., 2022). COVID-19 likely has a multifaceted impact, involving factors such as direct viral invasion of muscle tissue, systemic inflammation, and the prolonged immobility experienced during severe illness, particularly for those requiring hospitalization or mechanical ventilation (Soares et al., 2022). These factors may lead to muscle atrophy, alterations in muscle structure, and decreased neuromuscular efficiency, impairing functional capacity and delaying recovery (Soares et al., 2022; Andalib et al., 2021; Aschman et al., 2021).

Understanding the relationship between muscle structure and function is essential for unraveling the physiological foundations of force production (Lieber and Fridé, 2000), especially to better comprehend how COVID-19 can affect the musculoskeletal system. Muscle architecture is the primary determinant of muscle function and can be defined as the macroscopic arrangement of muscle fibers relative to the axis of force production (Lieber and Fridé, 2000). Therefore, assessing the fascicle length (FL) and pennation angle (PA) is critical to understanding the muscle architecture and, consequently, its force production capacity. FL is related to the number of sarcomeres in series, determining the fiber/muscle shortening velocity and excursion (Thom et al., 2007; Blazevich and Sharp, 2005; Abe et al., 2000; Cavalcante et al., 2021a). PA is associated with the parallel arrangement of sarcomeres within the fiber, dictating the maximum force production and transmission efficiency (Blazevich and Sharp, 2005; Cavalcante et al., 2021a; Fukunaga et al., 1997; de Jesus Ferreira et al., 2022). When comparing the architecture within the same muscle, greater force production is generally associated with larger PA, shorter FL, and increased cross-sectional area (CSA), reflecting a more optimized arrangement for force generation and transmission (Lieber and Fridé, 2000). Additionally, the stiffness of the tendon-aponeurosis complex (TAC) reflects the elongation of the deep aponeurosis to the distal free tendon in response to muscle force transmission to the bones (Cavalcante et al., 2021a; Raiteri et al., 2018; Burgess et al., 2009; Kub et al., 2006). In long-COVID cases, alterations in muscle strength, thickness, fatigue, and tendon properties can persist for years (Almeida et al., 2025; Ferreira et al., 2025). The majority of studies focus on hospitalized or recently discharged patients, relying on ultrasound assessments at rest (Damanti et al., 2022; Dams et al., 2024). However, research on active muscle architecture parameters, such as TAC displacement, PA, and FL, during both rest and maximal contraction is needed to determine the physiological adaptation resulting from long COVID-19.

The quadriceps femoris (QF) is critical for activities such as walking, climbing stairs, and standing up (Bordoni and Varacallo, 2023). COVID-19 significantly affects the QF, particularly by increasing fatigability in post-infection cases (Almeida et al., 2025). The properties of the QF constituents and the TAC reflect muscle and tendon architecture and can be used to assess muscle quality in both health and recovery from injury (Cavalcante et al., 2021a; de Jesus Ferreira et al., 2022; Cavalcante et al., 2021b; de Sousa et al., 2023). Changes in QF architecture and TAC properties may provide critical insights into the impact of COVID-19 on muscle and tendon function, as well as the healing process. Herein, in three cohorts of participants (moderate and severe cases of COVID-19 compared to a healthy control group), we aimed to A) assess the longitudinal effects of COVID-19 on the muscle architecture and TAC of the VL and RF over a one-year post-infection period and related inactivity, and B) investigate the relationship between torque production and these structural and mechanical properties, including TAC stiffness, FL, and PA. We hypothesized that severe COVID-19 would lead to more pronounced alterations in muscle architecture and TAC stiffness over time, including reduced PA, increased FL, and decreased TAC stiffness, compared to moderate COVID-19 and control groups.

## 2 Materials and methods

### 2.1 Design

This longitudinal observational study was conducted following approval from the Institutional Review Board at University of Brasília (CAAE 45043821.0.0000.8093) with a secondary outcome assessment registered at the clinical trial platform (NCT04961255). Informed consent was obtained from all participants prior to their inclusion in the study.

### 2.2 Participant selection

The study recruited participants aged 18–80 years. Participants with confirmed COVID-19 infection (based on PCR test results provided by certified laboratories) were divided into two groups according to disease severity. Severity classification was self-reported and based on clinical symptoms, hypoxemia, and hospitalization history, following the criteria described by Siddiqi and Mehra (2020): The Moderate COVID group consisted of participants with a confirmed positive COVID-19 test, (positive IgM and IgG antibodies). This group exhibited symptoms such as dry cough, runny nose, sore throat, body aches, and persistent fever, without hypoxemia, and did not require hospitalization. The Severe COVID group also tested positive for COVID-19 and presented with one or more of the aforementioned symptoms, along with hypoxemia that necessitated hospitalization, with or without the need for mechanical ventilation (Siddiqi and Mehra, 2020; Gandhi et al., 2020). The control group consisted of participants who had not been infected by the virus and/or did not exhibit any COVID-19 symptoms, and were included for comparative purposes.

### 2.3 Study procedure

The COVID-19 groups were recruited 21 days after the onset of symptoms, a period considered safe for transmission (Tenforde et al., 2020). These groups underwent four evaluations over a one-year period (Sigfrid et al., 2021): the first between days 21–30 (D_21-30_), the second between days 31–90 (D_31-90_), the third between days 91–180 (D_91-180_), and the final evaluation between days 181–360 (D_181-360_) after symptom onset or, in severe cases, hospital discharge. The control group was evaluated only once, as several participants later contracted COVID-19, making it unfeasible to continue evaluating them throughout the entire study duration. Demographic data and fatigue levels, measured using the 9-item Fatigue Severity Scale (FSS), were collected at the start of each assessment. The FSS rates agreement on a scale of 1 (strongly disagree) to 7 (strongly agree), with higher scores indicating greater fatigue severity. The final score is the mean of all item ratings (Krupp and Pollina, 1996).

### 2.4 Experiment outline

The participants were positioned and secured in an isometric dynamometer (Flexo/Extensor Chair, Cefise, Nova Odessa, SP). The device’s axis was carefully aligned with the flexion-extension axis of the participant’s knee, specifically at the lateral epicondyle of the femur (Nats et al., 2018). A goniometer was used to set the knee at 60° of flexion and the hip at 90° of flexion (Cavalcante et al., 2021a). The lever arm of the dynamometer’s power transducer was fastened securely with a strap placed 2–3 cm above the lateral malleolus. Resting torque values were measured and later adjusted to account for gravitational forces due to the limb’s weight or other factors, such as passive tension in structures crossing the knee joint (Cavalcante et al., 2021a). These angles were consistent across all sessions and participants. A familiarization session was conducted, consisting of 3–5 maximal voluntary isometric contractions performed in a ramp pattern. The participants were instructed to gradually increase force over approximately 5 s, until reaching their maximum effort. Verbal encouragement and visual feedback of the produced torque were provided to the participant during each MVIC, with a concomitant ultrasound system (M-Turbo®, Sonosite, Bothell, WA, USA) equipped with a wide-band linear array probe (40 mm, 7.5 MHz, depth 6.0 cm, acquisition frame of 30 Hz) for image acquisition. The probe was positioned longitudinally along the muscle fibers and perpendicular to the skin at specific anatomical locations at 50% (RF) and 60% (VL) from proximal to the distal, of the distance between the medial aspect of the anterior superior iliac spine and the patella base (Massey et al., 2018; Blazevich et al., 2006). These regions were preferred because an isotropic muscle architecture and minimal fascicle curvature were expected (Cavalcante et al., 2021a; Blazevich et al., 2006). With cine-loop ultrasound imaging, two recordings were made for each muscle, capturing the transition from rest to the maximum contraction. The recording that provided the clearest visualization of the fascicles was selected for further analysis.

### 2.5 VL and RF structural properties

With the participant positioned on the isometric dynamometer, the probe was aligned to view the parallel superficial and deep aponeuroses, and multiple fascicles were clearly visible within the image in the RF and VL (Baroni et al., 2013; Geremia et al., 2019). Frames from resting state and maximum contraction were saved as image files to analyze FL and PA, using ImageJ software (v.1.46; National Institutes of Health, Bethesda, United States). The best fascicle (defined as the one that could be distinctly traced from its insertion on the deep aponeurosis to the edge of the probe’s field of view) was selected for analysis (Geremia et al., 2019). The PA was considered as the angulation of the fibers concerning the muscle’s line of action of force, measured by assessing the angle formed between the muscle fiber and the deep aponeurosis (Lima and De Oliveira, 2013). The FL was considered as the total length of the muscle fiber. The FL portion, from the field-of-view boundary to the superficial aponeurosis, was estimated by equation according to previous studies (Blazevich and Sharp, 2005; Cavalcante et al., 2021a; Finni and Komi, 2002). As the ends of the fascicles were outside the ultrasound image, FL was estimated from extrapolation, as recommended in a previous study (Finni and Komi, 2002). The error in estimating the entire FL using the linear model ranged from 2% to 7% (Finni and Komi, 2002) to 13% (Noorkoiv et al., 2010). The region of interest (ROI) was identified and consistently maintained across all four time points using a plastic sheet with the outline from the first time point (Cavalcante et al., 2021a; Finni and Komi, 2002). All measurements were taken by a single investigator with substantial experience in ultrasonography, but the ultrasound videos and images were processed and analyzed by two other independent raters. For FL and PA at rest and at MVIC, three measurements were taken, and their average was used as the final value to ensure accuracy and reliability. A recent cornerstone methodological guideline was therefore adopted and adapted for the present study (Martin-Rodriguez et al., 2024), with the aim of ensuring anatomically consistent region-of-interest (ROI) selection and enhancing reproducibility across longitudinal assessments over the one-year follow-up period.

### 2.6 TAC displacement and TAC stiffness index

The displacement of the TAC for the VL and RF was measured from the same videos used to analyze the muscle architecture. A custom device stabilized the probe during torque production (Cavalcante et al., 2021a). Tracker 4.87 software was used to track the displacement of the fascicle relative to the deep aponeurosis, from rest to maximum contraction. To account for the possibility that the deep insertion of the fascicle was outside the transducer’s field of view, linear extrapolation was used (Massey et al., 2018; Ando et al., 2014). The force produced by the participant during each contraction was measured in Newtons (N) by the ratio of torque produced by the moment arm of the knee joint, set at 0.056 m for 60° of flexion, according to previous studies (Cavalcante et al., 2021a; Cavalcante et al., 2021b; de Sousa et al., 2023; Krevolin et al., 2004). To determine the TAC stiffness index, we used the delta from 40% to 100% of the MVIC and the delta of fascicle displacement for the VL and RF from 40% to 100% of maximum force (Cavalcante et al., 2021a). This range was chosen because, at this level of contraction, the musculotendinous unit is expected to be fully engaged, eliminating any slack (Cavalcante et al., 2021a). By focusing on this portion of the force-displacement curve, we ensured that the stiffness measurements reflected the true mechanical properties of the tendon-aponeurosis complex under active loading conditions (Cavalcante et al., 2021a). The overall stiffness index was obtained by averaging the force produced during the RF and VL videos and the average displacement of both muscles. Therefore, the TAC stiffness index = ([100% force - 40% force of RF + VL/2]/[TAC displacement of RF + VL/2]) (Cavalcante et al., 2021a). This stiffness index was used as an indirect measure of muscle-tendon force transmission, defined in this study as the capacity of the tendon-aponeurosis complex to transmit the force generated by the muscle fascicles to the skeletal system during contraction (Ma et al., 2010).

**FIGURE 1:**
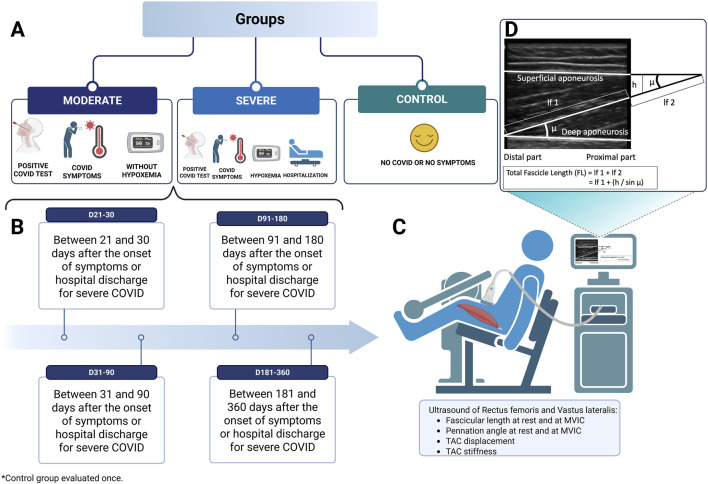
Study design, flow and testing protocol for muscle variable assessments at 60 degrees of knee flexion and 90 degrees of hip flexion. **(A)** groups; **(B)** assessments carried out over 1 year; **(C)** ultrasound assessment. **(D)** Fascicle length estimation–pennation angle (*µ*) corresponds to the angle between the deep aponeurosis and the fascicle. When the end of the fascicle extended off the acquired ultrasound image, fascicle length (*L*f) was estimated by trigonometry [total *L*f = *l*f 1 (measured fascicle length) + *l*f 2 (estimated fascicle length) = *l*f 1 + (*h*/sin *µ*), where *h* is height] by assuming a linear continuation of the fascicles. Adapted from Abellaneda et al. (2009) (Abellaneda et al., 1985). MVIC, maximal voluntary isometric contraction, TAC, tendon aponeurosis complex. Created with BioRender.com.

To synchronize torque with fascicle displacement captured by ultrasound, a LabChart Pro (AD Instruments, Dunedin, New Zealand) was connected to the isometric dynamometer. An HD webcam recorded the ultrasound screen, while a visual indicator marked the start of recording to ensure synchronization (Cavalcante et al., 2021a; Bojsen-Moøller et al., 2003). Figure 1 illustrates the study design flowchart, detailing the participant groups (Figure 1A), the timeline of assessments over the one-year follow-up period (Figure 1B), the content of each assessment (Figure 1C), and the fascicle length estimation (Figure 1D).

### 2.7 Statistical analysis

To analyze differences in muscle variables between and within the groups, the Generalized Estimating Equations (GEE) method was applied, considering “groups” (moderate COVID, severe COVID, and control) and “time” (D_21-30_, D_31-90_, D_91-180,_ and D_181-360_) as factors. The model was adjusted to incorporate “torque” as a covariable, aiming to determine whether torque affects the behavior of the observed variables. This statistical model, accounting for torque, was deemed the most suitable for all variables based on the quasi-likelihood under the independence model criterion (QIC), as suggested by Pan (2001), when compared to models including other factors, such as sex, age, and the body mass index (BMI). Post hoc analyses were performed using the least significant difference to identify specific differences.

Additionally, the GEE method with “group” as a factor was employed to compare continuous variables between the three groups. Categorical variables were compared using the Chi-square test. A significance level of α <0.05 was established, and all statistical analyses were carried out using SPSS 22.0 software (IBM Corporation, Armonk, NY, USA).

To determine inter-rater reliability for the analysis of ultrasound images and videos, the intraclass correlation coefficient (ICC) was calculated using an absolute agreement model and bidirectional mixed effects model. The magnitude of the ICC was interpreted using Mukaka’s classification (Mukaka, 2012): 0.00 to 0.30, insignificant; 0.31 to 0.50, low; 0.51 to 0.70, moderate; 0.71 to 0.90, high; and 0.91 to 1.00, very high. The Coefficient of Variation (CV) was provided for reliability analysis (mean ± standard deviation). For this, 20 participants from the study were randomly selected to determine the ICC.

## 3 Results

### 3.1 General characteristics of the participants

Table 1 summarizes the characteristics of the participants, allocated into three groups: 22 in the moderate-COVID group, 18 in the severe-COVID group, and 30 in the control group. Significant differences were observed between groups for age (p < 0.001), weight (p < 0.001), and BMI (p < 0.001), with the severe-COVID group being older and heavier. Furthermore, the severe-COVID group had a higher prevalence of comorbidities, including hypertension (p < 0.001), diabetes (p < 0.001), dyslipidemia (p = 0.021), and depression (p = 0.003). The severe-COVID groups presented significantly lower physical activity levels (p < 0.001) and lower knee extension torque (p = 0.007) compared to the moderate-COVID and control groups. Also, in the severe group, 38.89% of the participants required mechanical ventilation for an average of 7 days. None of the hospitalized participants were diagnosed with ICU-acquired weakness (ICUAW). Furthermore, both the severe and moderate groups showed persistently elevated levels of fatigue across all assessments when compared to the control group (p < 0.001).

**TABLE 1:** General characteristics of the study participants.

Characteristics	Groups			p
	Control	Moderate COVID	Severe COVID	
	Mean (CI 95%)	Mean (CI 95%)	Mean (CI 95%)	
Sex (n)				
Male	13	10	8	0.988
Female	17	12	10	
Age (years)				
	46.53 (42.10–51.43)	38.27 (33.96–43.13) *	50.83 (45.19–57.18)^#^	0.001
Weight at baseline (kg)				
	69.54 (65.07–74.31)	71.28 (64.14–79.21)	83.08 (75.91–90.92)^#^ *	0.006
Height (m)				
	1.66 (1.63–1.69)	1.66 (1.62–1.70)	1.61 (1.55–1.66)	0.206
BMI at baseline (kg/m^2^)				
	24.91 (23.85–26.02)	25.55 (23.51–27.77)	31.94 (29.70–34.35)^#^ *	0.001
Torque at baseline (N.m)				
D_21-30_	139.20 (125.15–154.84)	129.43 (111.12–150.77)	95.24 (74.93–121.06)^#^ *	0.007
D_31-90_		148.54 (127.17–173.49)^a^	105.96 (85.72–130.97)^#^ *	
D_91-180_		156.77 (132.66–185.25)^a^	108.07 (86.75–134.64)^a#^ *	
D_181-360_		152.00 (129.70–178.12)^a^	102.15 (85.40–122.17)^#^ *	
FSS				
D_21-30_	2.75 (2.33–3.24)	4.60 (4.09–5.16)*	5.52 (4.93–6.18)^#^ *	0.001
D_31-90_		3.68 (3.08–4.40)*^a^	4.47 (3.85–5.20)*^a^	
D_91-180_		3.77 (3.24–4.38)*^a^	4.47 (3.87–5.17)*^a^	
D_181-360_		3.59 (3.01–4.29)*^a^	4.78 (4.03–5.67)^#^ *	
Comorbidities (n)				
Hypertension	5	1	12^#^	0.001
Diabetes	0	1	7*	0.001
Dyslipidemia	6	3	9^#^	0.021
Depression	0	1	5*	0.003
Anxiety	1	5	3	0.102
Panic Syndrome	0	1	2	0.183
Asthma	0	4	2	0.062
Hospitalization period (days)				
	N.A	N.A	38.38 (27.15–49.62)	N.A
Period in ICU (days)				
	N.A	N.A	21.44 (13.72–29.16)	N.A
Need for mechanical ventilation (n)				
Yes	0	0	7	N.A
No	30	22	11	
Time of mechanical ventilation (days)	N.A	N.A	7.11 (12.12–2.10)	N.A
COVID vaccine before infection (n) for moderate and severe groups and COVID vaccine at the time of evaluation for control group (n)				
Yes	28	22	16	0.298
No	2	0	2	
Number of doses	3.50 (3.25–3.77)	2.14 (1.89–2.41) *	2.00 (1.72–2.32) *	0.001
Practice of physical activity before infection (n) and at the time of evaluation for control group^$^				
Yes	19	9	5*	0.045
No	11	13	13	
Practice of physical activity at the time of evaluation (n) ^$^				
D_21-30_				0.001
Yes	19	8	3 ^#^*	
No	11	14	15	
D_31-90_				
Yes		14	5 ^#^*	
No		8	13	
D_91-180_				
Yes		17	5 ^#^*	
No		5	13	
D_181-360_				
Yes		14	4 ^#^*	
No		8	14	
Physiotherapy after infection (n)				
Yes	NA	0	13^#^	0.001
No	NA	22	5	

Legend: NA, not applicable; CI, 95%, 95% confidence interval. FSS, fatigue severity scale questionnaire. D_21-30_, assessment carried out between 21 and 30 days after the onset of symptoms or hospital discharge for severe COVID, D_31-90_, assessment carried out between 31 and 90 days after the onset of symptoms or hospital discharge for severe COVID, D_91-180_, assessment carried out between 91 and 180 days after the onset of symptoms or hospital discharge for severe COVID, and D_181-360_, between 181 and 360 days after the onset of symptoms or hospital discharge for severe COVID., Control group evaluated only once. * = Different from the control group in the respective assessment, # = Different from the moderate COVID, group in the respective assessment, a = Different from D_21-30_ within group, b = Different from D_31-90_ within group, c = Different from D_91-180_ within group. (p < 0.05).

^$^For this study, participants who reported being involved in any sporting or conditioning activity, regularly practicing the modality at least twice a week, were considered to be practicing physical activity (Dasso, 2019).

### 3.2 Reliability of measurements

We obtained very high reliability for the measurement of the TAC displacement of VL (ICC = 0.99, CV = 0.50 ± 0.46%) and RF (ICC = 0.99, CV = 2.94 ± 1.77%), as well as for FL and PA (RF FL at rest: ICC = 0.99, CV = 2.87 ± 3.05%; RF FL at 100% of MVIC: ICC = 0.99, CV = 3.31 ± 3.42%; VL FL at rest: ICC = 0.98, CV = 3.12 ± 5.14%; VL FL at 100% of MVIC: ICC = 0.98, CV = 5.79 ± 7.31%; RF PA at rest: ICC = 0.97, CV = 3.70 ± 4.23%; RF PA at 100% of MVIC: ICC = 0.99, CV = 3.20 ± 4.32%; VL PA at rest: ICC = 0.93, CV = 3.80 ± 5.26%; VL PA at 100% of MVIC: ICC = 0.95, CV = 4.69 ± 8.40%).

### 3.3 TAC displacement and TAC stiffness index

No interaction between “group” and “time” was observed for RF TAC displacement (p = 0.397). However, a significant group effect was found, with the severe-COVID group exhibiting lower TAC displacement than the control group (p = 0.002). A similar pattern was noted for the VL, with the severe-COVID group also presenting lower displacement values compared to the control group (p = 0.016) (Supplementary Table S1). Torque did not significantly affect displacement for either the RF or VL (p = 0.183 and p = 0.613, respectively). Regarding the TAC stiffness index, no differences were found between groups, as there was no interaction between “group” and “time” (p = 0.517), nor were there any isolated effects of group (p = 0.796) or time (p = 0.435) (Supplementary Table S1). However, torque significantly impacted the TAC stiffness index (β = 0.007, p = 0.001), indicating that each 1 N m increase in torque corresponds to a 0.007 N/mm rise in the TAC stiffness index.

### 3.4 Muscle architecture

**FIGURE 2:**
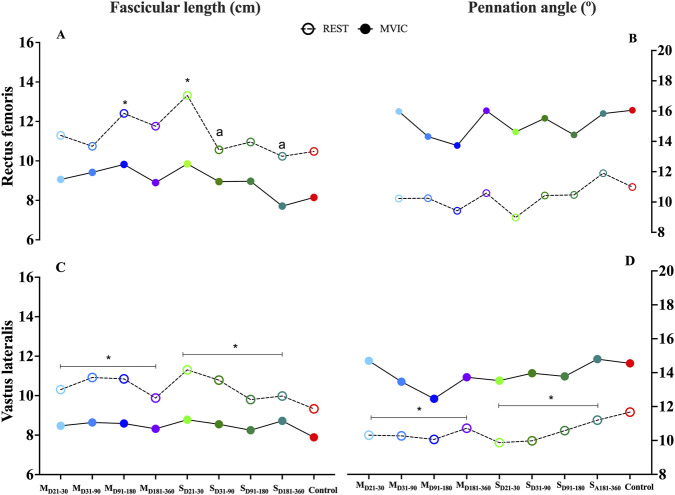
Muscle architecture changes in the rectus femoris and vastus lateralis at rest and during maximum voluntary isometric contraction (MVIC): Pennation angle (right y-axis) and fascicle length (left y-axis) of the rectus femoris and vastus lateralis at rest (dotted lines) and during MVIC (solid lines). Rectus femoris **(A,B)** and vastus lateralis **(C,D)**. Values expressed as mean. Legend: MVIC, maximum voluntary isometric contraction; M, moderate COVID group; S, severe COVID group. D_21-30_: assessment conducted between 21 and 30 days after symptom onset or hospital discharge for severe COVID; D_31-90_: assessment conducted between 31 and 90 days after symptom onset or discharge; D_91-180_: between 91 and 180 days; D_181-360_: between 181 and 360 days * = Different from the control group in the respective assessment, # = Different from the moderate COVID group in the respective assessment, a = Different from D_21-30_ within the group, b = Different from D_31-90_ within the group, c = Different from D_91-180_ within the group (p < 0.05).

When analyzing muscle architecture in both groups at rest and during maximal contraction (Supplementary Table S1), a significant interaction between “group” and “time” was observed for RF fascicle length (FL) at rest (p = 0.043). Post hoc analysis revealed that the severe-COVID group had longer FL during D_21-30_ compared to the control group (p = 0.016). Additionally, within the severe-COVID group, FL during D_21-30_ was significantly longer than at subsequent assessment times (D_31-90_: p = 0.014; D_181-360_: p = 0.034) (Supplementary Table S1; Figure 2A). Torque had no significant effect on FL at rest (p = 0.103). During MVIC, FL was similar between groups, with only a significant effect of ‘time’ (p = 0.025), showing shorter FL in the final assessment period, nearly one-year post-COVID (Supplementary Table S1). Torque also had no significant effect on FL during maximal contraction (p = 0.203). For the VL at rest, a significant group effect on FL was observed (p = 0.032), with the severe-COVID group exhibiting longer FL compared to the control group (p = 0.016). No significant torque effect was noted (p = 0.629). During maximal contraction, FL remained similar across all assessment times (p > 0.05), and torque did not significantly affect FL (p = 0.377) (Supplementary Table S1; Figure 2C).

For the RF, no significant group differences in PA were observed over time (p > 0.05). However, a significant association with torque was found (β = 0.001, p = 0.015), suggesting that participants with higher torque values tended to present greater resting PA. A similar pattern emerged during MVIC, with no group differences found over time (p > 0.05) (Supplementary Table S1; Figure 2B), yet torque remained significantly associated with PA (β = 0.001, p = 0.003), indicating that individuals producing higher torque generally exhibited greater PA during contraction. For the VL, resting PA showed no interaction between group and time (p = 0.278), but a significant group effect was noted (p = 0.012), with both COVID groups presenting smaller PA values compared to controls (moderate-COVID, p = 0.005; severe-COVID, p = 0.028). Again, torque was significantly associated with resting PA (β = 0.002, p = 0.001), meaning that participants with greater torque values also exhibited greater resting PA. During MVIC, although no significant group differences were observed across time points (p > 0.05), the association between torque and PA remained significant (β = 0.002, p = 0.001), reinforcing that individuals with higher torque tended to have higher PA values during maximal contraction (Supplementary Table S1; Figure 2D).

## 4 Discussion

The severe group exhibited reduced TAC displacement and lower QF torque compared to healthy controls, suggesting potential challenges in force transmission and a diminished ability of the TAC to stretch and recoil efficiently during muscle contractions. Despite these differences, torque appeared to influence TAC stiffness, suggesting that quadriceps force may partly explain the observed stiffness levels. Additionally, the severe group displayed longer RF fascicles at rest shortly after infection, while both COVID groups presented increased VL fascicle lengths at rest compared to controls. However, fascicle shortening during maximal contraction was similar across all groups, and the control group demonstrated larger PA in the VL at rest. Notably, higher torque levels were associated with greater PA in both resting and contracting conditions, reinforcing the relationship between muscle strength and architectural adaptations. These findings highlight the need for strength-focused rehabilitation strategies to mitigate long-term musculoskeletal deficits in post-COVID-19 recovery.

Different muscles exhibit distinct mechanical properties that determine their functional roles, with some favoring force generation and others prioritizing excursion (Lieber and Fridé, 2000). Muscle velocity and excursion are influenced by the number of sarcomeres in series, while muscle force is primarily determined by the CSA of sarcomeres and the fiber orientation relative to the deep aponeurosis (Lieber and Fridé, 2000). In our study, the severe COVID-19 group exhibited a longer FL in the RF immediately after infection, which suggests an atypical muscular adaptation. Typically, reduced mechanical loading and bed rest lead to a decrease in FL due to sarcomere loss in series (Narici and Cerretelli, 1998; Delp et al., 2000). However, our findings showed a transient increase in FL, followed by a gradual reduction over time. While the literature predominantly associates sarcomere addition with chronic stretching or immobilization in a lengthened position (Lieber and Fridé, 2000), there is limited evidence linking this response to systemic inflammation and viral infections (Straight et al., 2020; Deyhle et al., 2016; Tecer et al., 2023). A potential explanation for this is that severe COVID-19 triggers compensatory mechanisms aimed at preserving muscle function in response to neuromuscular deficits and strength loss (Thomas and Grumbles, 2014; Dambreville et al., 2016). Factors such as oxidative stress, prolonged inflammation, and altered neuromuscular control may have contributed to this adaptation, although the underlying mechanisms remain unclear. Given the lack of studies on the effects of long COVID-19 on muscle architecture, further longitudinal research comparing pre- and post-infection muscle changes is warranted to observe these modifications after 1 year of follow-up.

Regarding PA, our results reinforce its relationship with torque, observed both at rest and during MVIC. Trezise, Collier and Blazevich (2016) (57) have shown that PA plays a role in maximal force production. Specifically, a greater PA facilitates a higher packing density of muscle fibers, allowing for greater force generation and transmission, albeit at the cost of reduced shortening velocity. Consistently, the control group, which demonstrated higher torque values, also exhibited a greater PA in the VL at rest compared to both COVID-19 groups. However, while stronger muscles tend to present larger PA, this relationship should be interpreted alongside other factors, such as muscle CSA and neuromuscular activation, which also play critical roles in force generation (Trezise et al., 2016). Our results suggest that the reduced PA in the VL at rest in long COVID patients may reflect localized impairments in force generation and transmission, potentially due to fiber atrophy or changes in muscle composition. While this finding highlights potential muscle weakness and addresses our aims, it is important to consider that this change was observed in a single component of the quadriceps femoris and may not fully represent global knee extension strength. This underscores the need for targeted rehabilitation strategies, such as resistance training combined with neuromuscular electrical stimulation, that address both muscle-specific and global deficits.

In the current study, while the groups exhibited similar TAC stiffness, our analysis revealed a strong association between TAC stiffness and torque production, considering both the force generated and the fascicle shortening during contraction. Notably, the severe COVID-19 group, despite having comparable TAC stiffness, demonstrated both reduced TAC displacement and lower torque production. A possible explanation for the preserved TAC stiffness in the severe group lies in the way stiffness is calculated - through the ratio between force and displacement (Cavalcante et al., 2021a). Since both parameters were reduced in this group, their proportional relationship may have remained unchanged. Additionally, alterations in muscle architecture, such as increased FL and reduced PA, could have affected force transmission to the tendon, limiting its elongation under load. A lower PA limits the muscle’s ability to pack fibers obliquely, reducing the effective force transferred to the tendon. Meanwhile, a greater FL favors contraction velocity but may compromise force production, especially in isometric and eccentric contractions (Lieber and Fridé, 2000; Trezise et al., 2016). Moreover, a prolonged period of bed rest and ICU stay, which averaged 21 days in the severe group, may have led to neural activation deficits, further compromising muscle-tendon interaction (Duchateau, 1995). These findings highlight the importance of considering both structural and neuromuscular factors in post-COVID-19 rehabilitation, as preserved stiffness does not necessarily indicate preserved function.

In addition, the severe COVID-19 group exhibited distinct characteristics, including age, lower physical activity levels, a higher body mass index, reduced torque production, more comorbidities, and prolonged bed rest, all of which may have influenced our findings. Severe COVID-19 is strongly linked to risk factors, such as advanced age and comorbidities, which increase vulnerability (Croo et al., 2021; Wolff et al., 2021; Wu et al., 2020). A meta-analysis of 59 studies demonstrated that patients over 70 have a 65% higher risk of severe COVID-19 (Pijls et al., 2021). Furthermore, physical inactivity - defined as not meeting the World Health Organization’s recommended weekly physical activity levels (World Health Organization, 2010) raises the hospitalization risk by 30% (Hamer et al., 2020). These factors, collectively, exacerbate musculoskeletal deterioration. Aging reduces fiber size and number, CSA, PA, and FL (Narici and Maganaris, 2007), which are further compounded by bed rest (Blazevich and Sharp, 2005; Reeves et al., 2002) and sedentary behavior (Fitts et al., 1991). Therefore, it is crucial to recognize that COVID-19 represents a complex and multifactorial condition. The impact of COVID-19 on the musculoskeletal system is multifactorial, involving risk profiles, inactivity, and systemic inflammation, all contributing to muscle degradation.

The current study presents some limitations. First, it was not possible to collect longitudinal data from control participants over a one-year period because many of them contracted the virus during the study. Additionally, data collection took place during the height of the COVID-19 pandemic, a time characterized by strict social restrictions, heightened public health concerns, and widespread reluctance to participate in face-to-face research activities. These challenges significantly impacted the recruitment of a control group with baseline characteristics comparable to those of the COVID-19 groups. As a result, matching groups for variables like age, physical activity level, comorbidities, time of hospitalization in intensive care units and the use of mechanical ventilation may have introduced confounding factors. Particularly, the age range selected for evaluation in this study was intentionally broad to reflect the real-world circumstances of the pandemic at the time. This approach was necessary given recruitment constraints but may have inevitably affected our results by including individuals across a wide age spectrum, which could influence muscle-tendon outcomes due to age-related physiological differences. Similarly, physical activity levels and sedentary behavior were not directly measured during the follow-up period, which limits our ability to fully account for their influence on the outcomes. To partially address this limitation, we implemented the FSS at each time point. Although the FSS does not replace objective assessments of physical activity, it provided meaningful insights into participants’ perceived fatigue levels, particularly relevant in the context of post-COVID-19 recovery, where fatigue may reflect reduced physical functioning and engagement. Also, a formal power analysis was not conducted for this study due to the lack of available data on musculoskeletal outcomes in post-COVID-19 populations at the time of study design. Additionally, data collection took place during the peak of the COVID-19 pandemic, which presented significant challenges for participant recruitment. As a result, the sample size was limited, which may have reduced the statistical power to detect more subtle longitudinal effects or interactions. Regarding ultrasound analysis, we did not assess muscle CSA due to the lack of panoramic imaging on our ultrasound device, and echo intensity was excluded to avoid the risk of type I errors from multiple comparisons in our ultrasound analyses. Also, one of the main limitations of this study relates to the estimation of FL using a linear extrapolation method (Equation B) based on images acquired with a 40 mm ultrasound probe. While this method has been widely used in studies assessing quadriceps muscle architecture, including in longitudinal designs, it is subject to certain assumptions and potential sources of error. Previous literature has reported estimation errors ranging from approximately 2%–7% (Finni and Komi, 2002) to 13% (Noorkoiv et al., 2010), depending on the muscle assessed, probe positioning, and the extent of fascicle curvature. Although we implemented strict controls for probe placement using a standardized reference sheet to ensure consistent ROI alignment across all sessions (Cavalcante et al., 2021a), we acknowledge that this methodological choice may limit the precision of the absolute fascicle length values. Additionally, panoramic imaging or extended field-of-view (EFOV) approaches may provide more accurate estimates in future studies and are recommended where available. Also, ultrasound image acquisition is inherently subject to operator-dependent variability, which may influence muscle architecture measurements despite efforts to standardize the procedure. Importantly, our findings are limited to the specific muscles assessed in this study and, therefore, should not be generalized to other muscle groups. Nonetheless, the observed alterations provide valuable insights into post-COVID-19 muscular alterations and highlight the need for further investigations in this area, given the complexity of the impact on muscular health. Future longitudinal cohort studies extending over the next 5–10 years are recommended to better understand long-term outcomes. These studies could guide the development of physical activity programs aimed at restoring and maintaining muscle mass in individuals affected by COVID-19.

## 5 Conclusion

Participants with severe COVID-19 exhibited alterations in muscle architecture, including increased fascicle length and reduced pennation angle, which may contribute to persistent muscle weakness 1 year after infection. Additionally, the reduction in TAC stiffness index observed in these individuals suggests changes in the mechanical behavior of the muscle-tendon unit, although this was assessed through indirect estimations. These findings underscore the potential relevance of quadriceps strengthening strategies aimed at promoting architectural adaptations and improving mechanical efficiency in individuals recovering from severe COVID-19.

## Data Availability

The original contributions presented in the study are included in the article/Supplementary Material, further inquiries can be directed to the corresponding author.
