# Supplementary material for: Long COVID-19 alters muscle architecture and muscle-tendon force transmission: a one-year longitudinal study
Source: Front Physiol. 2025 Aug 25;16:1641046. doi: 10.3389/fphys.2025.1641046 (PMC12415051; doi:10.3389/fphys.2025.1641046)
Supplement: Supplementary file 1 [file Table1.docx]

Supplementary Material

# Supplementary Table 1

| **Supplementary table 1.** Muscle variables in patients who were infected with moderate and severe forms of COVID-19 compared to a control group. | | | | | | | | | |
| --- | --- | --- | --- | --- | --- | --- | --- | --- | --- |
| Muscle variables |  | D_21-30_ | D_31-90_ | D_91-180_ | D_181-360_ | General (All assessments) | GEE (p values) | | |
|  | Groups | Mean (CI 95%) | Mean (CI 95%) | Mean (CI 95%) | Mean (CI 95%) | Mean (CI 95%) | Group | Assessment | Group * Assessment |
| Displacement RF (mm) | Control | 22.74 (20.68 – 25.01) |  |  |  | 22.74 (20.68 – 25.01) | 0.008 | 0.635 | 0.397 |
|  | Moderate | 20.16 (16.18 – 25.10) | 20.41 (16.30 – 25.56) | 21.23 (18.34 – 24.56) | 17.74 (15.07 – 20.89) | 19.84 (17.09 – 23.03) |  |  |  |
|  | Severe | 18.16 (15.40 – 21.42) | 17.85 (15.22 – 20.94) | 17.54 (14.94 – 20.59) | 18.09 (15.73 – 20.80) | 17.91 (15.87 – 20.22) * |  |  |  |
|  | Total Sample | 20.27 (18.39 – 22.33) | 20.24 (18.39 – 22.27) | 20.38 (18.87 – 22.02) | 19.40 (17.98 – 20.93) |  |  |  |  |
| Displacement VL (mm) | Control | 22.89 (20.55 – 25.50) |  |  |  | 22.89 (20.55 – 25.50) | 0.047 | 0.843 | 0.868 |
|  | Moderate | 21.63 (16.79 – 27.88) | 20.12 (16.39 – 24.70) | 19.66 (15.92 – 24.27) | 19.67 (17.00 – 22.75) | 20.25 (17.13 – 23.94) |  |  |  |
|  | Severe | 18.45 (14.72 – 23.14) | 19.93 (16.86 – 23.56) | 19.07 (15.93 – 22.83) | 17.87 (15.54 – 20.56) | 18.82 (16.75 – 21.14) * |  |  |  |
|  | Total Sample | 20.91 (18.58 – 23.52) | 20.94 (19.11 – 22.94) | 20.47 (18.58 – 22.56) | 20.04 (18.61 – 21.58) |  |  |  |  |
| TAC Stiffness Index (N/mm) | Control | 50.72 (44.57 – 57.71) |  |  |  | 50.72 (44.57 – 57.71) | 0.796 | 0.435 | 0.517 |
|  | Moderate | 49.04 (41.65 – 57.73) | 54.13 (43.44 – 67.45) | 50.90 (43.09 – 60.12) | 52.66 (45.21 – 61.33) | 51.65 (41.86 – 55.87) |  |  |  |
|  | Severe | 51.67 (41.56 – 64.24) | 53.70 (42.85 – 67.29) | 43.82 (38.49 – 49.88) | 44.98 (39.53 – 51.17) | 48.36 (41.86 – 55.87) |  |  |  |
|  | Total Sample | 50.46 (45.69 – 55.74) | 52.83 (47.21 – 59.11) | 48.36 (44.60 – 52.44) | 49.34 (45.62 – 53.36) |  |  |  |  |
| RF fascicular length at rest (cm) | Control | 10.49 (9.60 – 11.47) |  |  |  | 10.49 (9.60 – 11.47) | 0.239 | 0.039 | 0.043 |
|  | Moderate | 11.29 (10.12 – 12.59) | 10.76 (9.81 – 11.80) | 12.45 (10.94 – 14.18) * | 11.76 (10.62 – 13.02) | 11.55 (10.87 – 12.27) |  |  |  |
|  | Severe | 13.32 (11.33 – 15.67) * | 10.52 (9.34 – 11.85) a | 10.96 (9.70 – 12.38) | 10.23 (8.93 – 11.72) a | 11.20 (10.39 – 12.07) |  |  |  |
|  | Total Sample | 11.64 (10.81 – 12.53) | 10.59 (9.98 – 11.23) | 11.27 (10.55 – 12.04) | 10.81 (10.14 – 11.52) |  |  |  |  |
| RF fascicular length at 100% of MVIC (cm) | Control | 8.15 (7.45 – 8.91) |  |  |  | 8.15 (7.45 – 8.91) | 0.092 | 0.025 | 0.074 |
|  | Moderate | 9.06 (7.81 – 10.50) | 9.42 (8.26 – 10.76) | 9.82 (8.64 – 11.15) | 8.90 (8.05 – 9.84) | 9.29 (8.65 – 9.99) |  |  |  |
|  | Severe | 9.85 (8.57 – 11.33) | 8.95 (7.80 – 10.28) | 8.97 (8.08 – 9.97) | 7.71 (6.92 – 8.59) | 8.84 (8.04 – 9.72) |  |  |  |
|  | Total Sample | 8.99 (8.34 – 9.70) | 8.83 (8.22 – 9.47) | 8.95 (8.42 – 9.52) | 8.24 (7.78 – 8.73) a,b,c |  |  |  |  |
| RF pennation angle at rest (º) | Control | 10.99 (10.00 – 12.08) |  |  |  | 10.99 (10.00 – 12.08) | 0.395 | 0.084 | 0.192 |
|  | Moderate | 10.23 (8.89 – 11.77) | 10.25 (9.03 – 11.62) | 9.42 (8.46 – 10.49) | 10.59 (9.26 – 12.11) | 10.11 (9.35 – 10.94) |  |  |  |
|  | Severe | 8.98 (7.21 – 11.18) | 10.43 (8.82 – 12.34) | 10.47 (9.32 – 11.75) | 11.90 (10.31 – 13.73) | 10.39 (9.34 – 11.57) |  |  |  |
|  | Total Sample | 10.03 (9.16 – 10.99) | 10.55 (9.78 – 11.38) | 10.27 (9.67 – 10.91) | 11.15 (10.38 – 11.97) |  |  |  |  |
| RF pennation angle at 100% of MIVC (º) | Control | 16.06 (14.49 – 17.78) |  |  |  | 16.06 (14.49 – 17.78) | 0.520 | 0.208 | 0.190 |
|  | Moderate | 15.98 (14.52 – 17.58) | 14.32 (12.73 – 16.12) | 13.73 (11.92 – 15.81) | 16.03 (14.41 – 17.84) | 14.98 (14.02 – 16.01) |  |  |  |
|  | Severe | 14.63 (12.39 – 17.27) | 15.53 (13.31 – 18.13) | 14.43 (12.81 – 16.25) | 15.84 (13.80 – 18.19) | 15.10 (13.58 – 16.79) |  |  |  |
|  | Total Sample | 15.54 (14.46 – 16.70) | 15.29 (14.23 – 16.42) | 14.71 (13.73 – 15.75) | 15.98 (14.95 – 17.08) |  |  |  |  |
| VL fascicular length at rest (cm) | Control | 9.33 (8.67 – 10.04) |  |  |  | 9.33 (8.67 – 10.04) | 0.032 | 0.170 | 0.052 |
|  | Moderate | 10.31 (9.37 – 11.35) | 10.92 (9.98 – 11.94) | 10.85 (9.90 – 11.90) | 9.88 (9.06 – 10.77) | 10.48 (9.86 – 11.15) * |  |  |  |
|  | Severe | 11.31 (9.85 – 13.00) | 10.79 (9.76 – 11.93) | 9.80 (8.87 – 10.83) | 9.98 (9.08 – 10.98) | 10.45 (9.70 – 11.27) * |  |  |  |
|  | Total Sample | 10.29 (9.67 – 10.94) | 10.35 (9.81 – 10.86) | 9.97 (9.48 – 10.49) | 9.73 (9.27 – 10.21) |  |  |  |  |
| VL fascicular length at 100% of MVIC (cm) | Control | 7.89 (7.32 – 8.50) |  |  |  | 7.89 (7.32 – 8.50) | 0.236 | 0.943 | 0.890 |
|  | Moderate | 8.47 (7.45 – 9.64) | 8.64 (7.92 – 9.43) | 8.59 (7.97 – 9.27) | 8.32 (7.63 – 9.08) | 8.51 (8.01 – 9.03) |  |  |  |
|  | Severe | 8.78 (7.64 – 10.10) | 8.55 (7.60 – 9.62) | 8.25 (7.35 – 9.27) | 8.72 (7.77 – 9.80) | 8.57 (7.78 – 9.45) |  |  |  |
|  | Total Sample | 8.37 (7.82 – 8.97) | 8.35 (7.91 – 8.83) | 8.24 (7.82 – 8.68) | 8.30 (7.87 – 8.76) |  |  |  |  |
| VL pennation angle at rest (º) | Control | 11.67 (10.89 – 12.51) |  |  |  | 11.67 (10.89 – 12.51) | 0.012 | 0.161 | 0.278 |
|  | Moderate | 10.31 (9.55 – 11.14) | 10.27 (9.31 – 11.32) | 10.06 (9.26 – 10.92) | 10.72 (9.90 – 11.61) | 10.34 (9.84 – 10.86) * |  |  |  |
|  | Severe | 9.87 (8.91 – 10.94) | 9.98 (9.21 – 10.81) | 10.58 (9.51 – 11.76) | 11.20 (9.96 – 12.60) | 10.39 (9.63 – 11.21) * |  |  |  |
|  | Total Sample | 10.59 (10.10 – 11.12) | 10.61 (10.12 – 11.13) | 10.75 (10.23 – 11.29) | 11.19 (10.63 – 11.79) |  |  |  |  |
| VL pennation angle at 100% of MIVC (º) | Control | 14.55 (13.40 – 15.80) |  |  |  | 14.55 (13.40 – 15.80) | 0.332 | 0.211 | 0.248 |
|  | Moderate | 14.70 (12.91 – 16.75) | 13.47 (12.29 – 14.76) | 12.46 (11.50 – 13.49) | 13.73 (12.51 – 15.06) | 13.56 (12.89 – 14.28) |  |  |  |
|  | Severe | 13.53 (12.11 – 15.12) | 13.97 (12.41 – 15.73) | 13.78 (12.45 – 15.25) | 14.80 (12.96 – 16.88) | 14.01 (12.75 – 15.40) |  |  |  |
|  | Total Sample | 14.25 (13.38 – 15.18) | 13.99 (13.22 – 14.81) | 13.57 (12.90 – 14.26) | 14.35 (13.52 – 15.23) |  |  |  |  |
| Legend: GEE, Generalized estimating equations. CI 95%, 95% confidence interval. D_21-30_, assessment carried out between 21 and 30 days after the onset of symptoms or hospital discharge for severe COVID, D_31-90_, assessment carried out between 31 and 90 days after the onset of symptoms or hospital discharge for severe COVID, D_91-180_, assessment carried out between 91 and 180 days after the onset of symptoms or hospital discharge for severe COVID, and D_181-360_, between 181 and 360 days after the onset of symptoms or hospital discharge for severe COVID. MIVC, maximal voluntary isometric contraction. RF, rectus femoris. VL, vastus lateralis. Control group evaluated only once. Moderate COVID (n=22); Severe COVID (n=18); Control (n=30); Total sample (n=70). *= Different from the control group in the respective assessment, #= Different from the moderate COVID group in the respective assessment, a= Different from D_21-30_ within group, b= Different from D_31-90_ within group, c= Different from D_91-180_ within group. (p<0.05). | | | | | | | | | |
